# Interplay Between Autophagy and Wnt/β-Catenin Signaling in Cancer: Therapeutic Potential Through Drug Repositioning

**DOI:** 10.3389/fonc.2020.01037

**Published:** 2020-08-18

**Authors:** Carlos Pérez-Plasencia, Eduardo López-Urrutia, Verónica García-Castillo, Samuel Trujano-Camacho, César López-Camarillo, Alma D. Campos-Parra

**Affiliations:** ^1^Laboratorio de Genómica Funcional, Unidad de Biomedicina, Facultad de Estudios Superiores Iztacala, UNAM, Tlalnepantla, Mexico; ^2^Laboratorio de Genómica, Instituto Nacional de Cancerología (INCan), Mexico City, Mexico; ^3^Posgrado en Ciencias Genómicas, Universidad Autónoma de la Ciudad de México, Mexico City, Mexico

**Keywords:** drug repositinging, autophagy, Wnt/b-catenin, cancer, signaling/signaling pathways

## Abstract

The widespread dysregulation that characterizes cancer cells has been dissected and many regulation pathways common to multiple cancer types have been described in depth. Wnt/β-catenin signaling and autophagy are among these principal pathways, which contribute to tumor growth and resistance to anticancer therapies. Currently, several therapeutic strategies that target either Wnt/β-catenin signaling or autophagy are in various stages of development. Targeted therapies that block specific elements that participate in both pathways; are subject to *in vitro* studies as well as pre-clinical and early clinical trials. Strikingly, drugs designed for other diseases also impact these pathways, which is relevant since they are already FDA-approved and sometimes even routinely used in the clinic. The main focus of this mini-review is to highlight the importance of drug repositioning to inhibit the Wnt/β-catenin and autophagy pathways, with an emphasis on the interplay between them. The data we found strongly suggested that this field is worth further examination.

## Introduction

The realization that cancer can be seen as a complex group of diseases rather than a unique one opened up the possibilities for different approaches to its treatment since it was clear that there is not a single, decisive event that leads to tumor formation (1–3) Instead, tumor development is the result of the cumulative and simultaneous deregulation of a significant number of cellular processes. Active research has generated a great deal of information on cellular events that underlie many of the Hallmarks of Cancer, and hence, are common to several tumors (4). These metabolic or signaling pathways are, thus, ideal targets for targeted therapy. As the name suggests, targeted therapies interfere with specific proteins involved in tumorigenesis, and promises to be more beneficial than conventional chemotherapy (5). The disadvantages of designing new targeted therapies are the elevated costs, the time it takes (6–9 years), and the need for three clinical trial phases before approval for patient use (5). As an alternative, drug repositioning has been considered, it refers to a new medical indication for an old drug that may currently be approved for another medical use (6). The advantages of this alterative are the reduction or elimination of drug development costs and the availability of comprehensive data on their pharmacology, formulation, safety and adverse effects (7). Potential disadvantages include handling patents, intellectual property, investment, market demand and even production technology (8). Nonetheless, drug repositioning has scope for consideration in cancer treatment (6).

Recent evidence suggests that Wnt/β-catenin signaling pathway and autophagy have an important role in cancer. Moreover, both pathways are oppositely regulated, since they are tightly interwoven through common regulation mechanisms (9–11). We reviewed the published literature looking for therapeutic strategies that blocked Wnt/β-catenin and autophagy; we found several reports of targeted therapies and repositioned drugs that attack both pathways individually, and that each pathway is regulated by the other. Interestingly, a substantial proportion of the drugs that we found were developed for treating diseases other than cancer and were repurposed with positive results, supporting this promising approach (12). We found it notable that drug repositioning grows increasingly advantageous.

## Wnt/β-Catenin Signaling Pathway in Cancer

The Wnt/β-catenin pathway is essential during development, regeneration, and cellular homeostasis (9). In a nontumoral context, Wnt ligands activate it by binding to the FZ/LRP5/6 receptor; this inhibits the formation of the destruction complex, comprised by Axin, APC, CK1, and GSK3. Lack of the destruction complex leads to the accumulation of cytoplasmic β-catenin and its translocation to the nucleus, where it binds TCF/LEF sites to activate transcription of target genes such as CCND1 and MYC, which are involved in cell proliferation and survival. In the absence of Wnt ligands, cytoplasmic β-catenin is phosphorylated by the destruction complex and targeted for proteasomal degradation by E3 ubiquitin ligases (13).

In cancer, Wnt/β-catenin signaling pathway is severely dysregulated in solid and non-solid tumors, being part of the proliferation pathways that characterize cancer cells (14). Although conducted by different mechanisms, this dysregulation is constant across several cancer types. For instance, in CRC it is driven by mutations in the tumor suppressor gene APC, These driver mutations favor the constitutive activation of Wnt/β-catenin signaling pathway, leading to the up-regulation of cyclin D1 and Myc, essential proteins involved in cell proliferation and cell-cycle progression (15). Wnt/β-catenin dysregulation has also been reported in cervical cancer (CC) (16). It is well known that Human Papillomavirus (HPV) is the etiological agent for cervical cancer, but Wnt/β-catenin dysregulation is recognized a “second hit.” In CC has been described only 20% of the analyzed cases displayed mutations (16). Besides, the antagonists of this pathway are decreased while positive components are increased, such as Wnt ligands (Wnt1, Wnt10), receptors (LRP6 FZD), and transducers (DVL and TCF4) (17). Also, in non-solid tumors such as leukemia, Wnt/β-catenin is dysregulated through increased expression of Frizzled-4, a receptor of WNT ligands (18, 19). Thus, this pathway, common to many cancers, is an attractive main target in cancer therapy.

## Autophagy in Cancer

Autophagy is a normal physiological process that mediates intracellular component degradation to maintain cellular homeostasis. This process initiates with the formation of a phagophore—a double membrane compartment—induced by the ULK1 and ATG proteins, which are, in turn, regulated by the AKT, AMPK, and mTOR pathways. The phagophore elongates and seals enclosing the cellular components to be degraded, thus generating a mature autophagosome; Beclin1, VPS34, and Atg14L are critical for this step. Then, the autophagosome fuses with endosomes and lysosomes to degrade the cellular material it has engulfed (11).

Autophagy was thought as a non-selective process elicited in response to starvation; however, it is now clear that degradation of protein aggregates, damaged or superfluous organelles, and the abolition of intracellular pathogens, are strongly regulated processes that require cargo recognition by the core ATG autophagy machinery, selective autophagy receptors and specificity adapters (20).

The selectivity of autophagy is mediated by receptors, which recognize cargos labeled with degradation signals, and by the autophagosomal membrane through its LC3- interacting regions (LIR), leading to the complete engulfment of damaged organelles by the autophagic membranes and subsequent degradation by ubiquitin-dependent or independent, selective autophagy pathways (20, 21). In ubiquitin-dependent autophagy, the substrates are targeted for degradation by interaction with P62, also known as sequestosome, which is associated with the proteasome (22).

The role of autophagy in cancer is still controversial, there is evidence suggesting that autophagy can cooperate in eliminating tumors or can promote tumor development, albeit not on every tumor stage. In early stages, autophagy acts as a tumor suppressor, limiting the proliferation of tumor cells, while, after the tumor is formed, the response to stress can raise the survival to increase metabolic requirements that are indispensable for tumor survival and rapid proliferation. Thus, autophagy is a promoter of advanced cancer and is well known to mediate resistance to several anticancer drugs by protecting cells from apoptosis (23). For instance, increased autophagy induced resistance to trastuzumab in breast cancer cell lines (24). Also, the repression of autophagy enhances the effect of 5-FU and cisplatin in colorectal and esophageal cancer cell lines (25, 26). Several findings have demonstrated that the accumulation of P62 aggregates impaired autophagy in several types of cancer (27). Likewise, ULK1 overexpression has been associated with colon, breast, lung, nasopharyngeal, and esophageal cancer. Indeed, ULK1 knockdown increases apoptosis and sensitizes lung cancer cells to cisplatin (28) Beclin 1 overexpression favored the resistance to gemcitabine-induced apoptosis in osteosarcoma cells (29); while its downregulation was associated with brain cancer, breast carcinoma, and prostatic carcinoma (30). Also, mutations in ATG genes were reported in colorectal carcinomas (31). Thus, autophagy is altered in cancer.

## Interplay Connecting Autophagy With Wnt/β-Catenin Signaling

**Figure 1:**
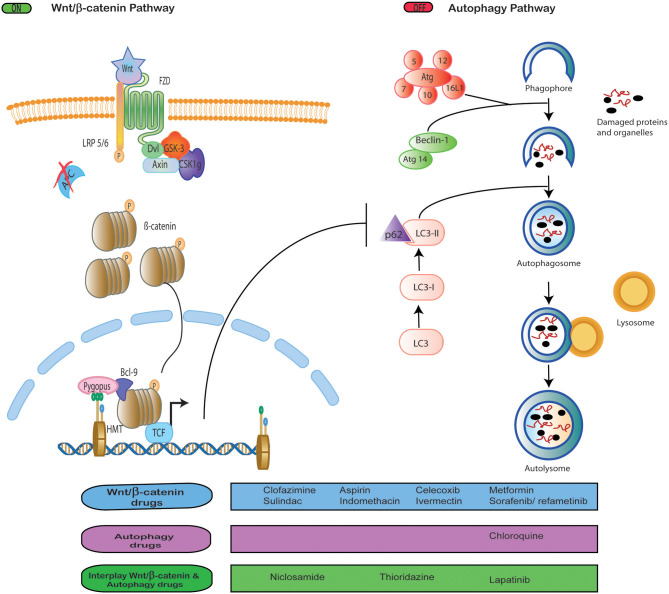
Interplay between Wnt signaling and autophagy. Drug repositioning to block Wnt/β-catenin signaling and autophagy, both independently and simultaneously.

Wnt/β-catenin signaling and autophagy pathways play essential roles during tumor development. Both pathways crosstalk with others such as NFKB (20), notch signaling (21), the ubiquitin-proteasome system (32), and DNA repair (33). However, we were interested in addressing the crosstalk between autophagy and Wnt/β-catenin signaling since the growing evidence points at the wide interrelation between both pathways, as discussed below (Figure 1).

Several reports note that Wnt/β-catenin activation is a negative regulator of autophagy based on the evidence that high levels of autophagy are incompatible with cell proliferation and survival promoted by Wnt/β-catenin (10). For instance, Thao et al. reported that the activation of Wnt/β-catenin inhibited Beclin 1 expression an element important to autophagic flux (29). Conversely, it has been reported that autophagy activation was able to downregulate Wnt/β-catenin signaling by degrading Dvl (34) or β-catenin (10). In colorectal tumors, the level of autophagy is inversely correlated with the activation of Wnt/β-catenin (35). In glioblastoma, multiple myeloma, and mammary tumors, it was observed that inhibition of Wnt/β-catenin pathway upregulates P62, LC3, and Beclin expression, and, in consequence, the autophagic flux (36–38). In ovarian cancer, it was demonstrated that DACT1 protein inhibit Wnt/β-catenin signaling and activate autophagy (39). While, in lung cancer, WIF-1 protein induced autophagy and inhibit Wnt/β-catenin signaling (40).

The involvement of WNT/β-catenin signaling in the regulation of autophagy was demonstrated in prostate cancer through the use of rapamycin and IWP-2, an inductor of autophagy and inhibitor of Wnt signaling, respectively (41). The reduction of β-catenin sensitized leukemia cell lines to the autophagy-stimulating mTOR inhibitors such as temsirolimus and rapamycin (42). Chloroquine inhibited autophagy, reverting the resistance to paclitaxel in lung cancer cells through modulation of the Wnt/β-catenin pathway (43).

There is *in vitro* evidence that the Wnt/β-catenin pathway and autophagy can be activated at the same time. Fan and collaborators reported that Wnt/β-catenin signaling was involved in autophagy-induced glycolysis in hepatocarcinoma cell lines. (44) Jing and colleagues found that the Wnt3a ligand stimulated autophagy through Wnt signaling in squamous cell carcinoma of the head and neck, sensitizing cells to radiotherapy (45). Together, this information about the role of Wnt/β-catenin and autophagy shows that, while both are valuable targets for therapies, Wnt/β-catenin signaling seems to downregulate autophagy in most tumor scenarios.

## Drugs That Target Wnt/β-Catenin Signaling

As mentioned before, the Wnt/β-catenin pathway is dysregulated in the majority of human cancers, so it is a promising therapeutic target; however, no targeted therapy has been approved for blocking WNT pathway to date. Only a few of them have made it into Phase I clinical trials, such as Ipafricept and vantictumab (WNT antibodies), LGK974 and ETC-159 (PORN inhibitors), PRI-724 and CWP232291 (β-catenin inhibitors) (46).

Drug repositioning becomes important here. Drugs approved for other diseases have shown the ability to block Wnt/β-catenin signaling and are candidates for being repurposed toward treatment for Wnt-dependent cancer, based on recent research. For instance, Ahmed and collaborators explored, in triple-negative breast cancer-derived cell lines and xerographs, the effect of clofazimine (approved to treat leprosy). This drug inhibited Wnt/β-catenin signaling efficiently, suppressed growth tumor, and induced apoptosis. Moreover, the authors observed that clofazimine was compatible with doxorubicin and that this combination demonstrated an additive effect both *in vitro* and *in vivo* (47).

Nonsteroideal anti-inflammatoty drugs (NSAIDs), are drugs promoted for their analgesic, anti-inflammatory, and anti-pyretic effects. Among them, it was reported that sulindac increased β-catenin degradation in breast, lung and colon cancer cells *in vitro*, through inhibition of the DVL-PDZ domain interaction (48, 49) Moreover, this drug reduced tumor growth in mouse models of metastatic colorectal cancer (50). Aspirin generated tumor reduction associated with lowered β-catenin in colorectal cancer murine models (51). Indomethacin—a Wnt/β-catenin signaling inhibitor—disrupts β-catenin-TCF4 in colorectal cancer, as demonstrated *in vitro* (52). Besides, Indomethacin was able to reduce tumor formation in rat models (53). NSAIDs as celecoxib inhibit cell proliferation by downregulating Wnt/β-catenin signaling in glioblastoma, prostate cancer, colon cancer, hepatoma and osteosarcoma (54–57).

Ivermectin, an antiparasitic drug, inhibits the expression of WNT-TCF targets, in colon cancer, glioblastoma, and melanoma xenograph models, and in and breast, skin, lung and intestine cell lines (58). Metformin, an antidiabetic drug, inhibits Wnt/β-catenin signaling in lung, pancreatic and gastric cancer cell lines, as well as in preclinical models of hepatocellular carcinoma and ovarian cancer (59). Sorafenib is a multikinase inhibitor and refametinib is a MEK inhibitor; both of them, in combination, repressed Wnt/β-catenin signaling, inhibiting cell proliferation and promoting apoptosis in hepatocellular carcinoma (HCC) models (60). Repurposed drugs that block Wnt/β-catenin signaling are solid candidates for WNT-dependent cancer treatment (Table 1).

**Table 1:** Targeted therapy and repositioning drugs to block Wnt/ β-catenin and autophagy.

**Targeted therapy to Wnt/β-catenin pathway**	**Cancer Type**	**References**
Ipafricept, vantictumab (WNT antibodies)	Ovarian cancers patients	(46)
LGK974, ETC-159 (PORN inhibitors)	Breast, head neck, melanoma, and pancreatic cancers patients	(46)
PRI-724, CWP232291 (β-catenin inhibitors)	Pancreatic cancers • Acute myeloid leukemia patients	(46)
**Repositioning drugs to block Wnt/β-catenin pathway**		
Clofazimine	Breast cancer cell lines and xerographs	(47)
Sulindac	Breast, lung, and colon cells	(48, 49)
Aspirin	Colorectal cancer murine models	(51)
Indomethacin	Colorectal cancer cells	(53)
Celecoxib	Glioblastoma, prostate cancer, colon cancer, hepatoma, and osteosarcoma cells	(54–57)
Ivermectin	Colon cancer, glioblastoma, and melanoma xenograph models and breast, skin, lung and intestine cell lines	(58)
Metformin	Lung, pancreatic, and gastric cancer cell lines and preclinical models of hepatocellular carcinoma and ovarian cancer	(59)
Sorafenib/ refametinib	hepatocellular carcinoma cells	(60)
**Repositioning drugs to target autophagy**		
Chloroquine	Pancreatic, colon, and renal cell carcinoma cancer patients	(62, 63)
**Targeted therapy to block autophagy**		
SBI-0206965	Leukemia cell lines	(65)
SAR405	Renal and lung cancer cancer cells	(67)
NSC185058	Osteosarcoma mouse models	(68)
SB02024	Breast cancer cells and xenographs	(69)
**Drugs to target interplay autophagy/Wnt/β-catenin pathway**		
Niclosamide	Colorectal cancer cancer cells	(71)
Thioridazine	Glioblastoma cells	(73)
Lapatinib	Cutaneous squamous cell carcinoma cells	(75)

## Drugs That Target Autophagy

Drug repositioning has also been applied to block the autophagy in cancer, using chloroquine. Chloroquine and its derivatives were originally approved to treat malaria and rheumatic disorders, but have been found to have an anticancer effect as well (61). Xu et al. reported a first meta-analysis, analyzing 7 clinical trials that evaluated chloroquine and hydroxychloroquine as autophagy inhibitors in cancer patients, the results showed that chloroquine-based therapy has better that other anti cancer treatments (62). Besides, phase I/II clinical trials that use this drug in combination with others such as temozolamide, vorinostat, everolimus, sorafenib, and gemcitabine to treat patients with pancreatic cancer, colon cancer, and renal cell carcinoma; the results show that these drug combinations are well tolerated, which raises opportunities for the benefit of patients (63).

Targeted therapies to block autophagy have been proposed; however, these have only been studied on cell lines and mouse models, not in patients as the repurposed drug chloroquine (64). For example, SBI-0206965, an ULK1 inhibitor explored in leukemia cell lines, was proposed for future exploration (65). ULK1/2 inhibitors MRT67307 and MRT68921, were tested only in fibroblast cells (66). SAR405 is a potent and selective Vps34 kinase inhibitor that synergized with everolimus to block proliferation of renal and lung cancer cells (67). The autophagy inhibitor NSC185058—an ATG4B inhibitor—demonstrated antitumor potential when tested in osteosarcoma mouse models (68) SB02024 is an VPS34 inhibitor tested in xenographs and breast cancer-derived cell lines that increases the effect of Sunitinib and Erlotinib (69). These targeted therapies are potential candidates to treat autophagy-dependent cancer; however, not until the needed clinical trials are performed (Table 1).

## Drugs That Target Wnt/β-Catenin and Autophagy Pathways

Although there are drugs that inhibit Wnt/β-catenin and autophagy individually, the interplay between both pathways is an attractive therapeutic target with a broader reach. As can be appreciated below, there are drugs that tackle cancer development through the disruption of the balance between these pathways. As evident in the preceding sections, where targeted therapies that block Wnt/β-catenin are just in early clinical trials and targeted therapies for autophagy are not even in that stage, the development of new drugs is complicated and requires many phases of study, thus several groups have resorted to drug repurposing to address this issue.

In this regard, it has been reported that niclosamide, a drug used originally to treat tapeworm infections and approved for FDA in 1982, is a multifunctional drug that has been demonstrated have an antitumor effect through several mechanisms (70). One of them is the inhibition of Wnt/β-catenin signaling by inducing autophagosomes (40). The detailed mechanism of action of niclosamide against cancer was recently reported, it stimulates the co-localization of Fzd1 or β-catenin with LC3 to their degradation by autophagosomes in colorectal cancer lines. This suggested LC3 as a biomarker to select patients who could receive this drug in future clinical trials and raised the need for further investigations on niclosamide-induced autophagy (71).

Thioridazine was approved as an antipsychotic drug more than 40 years ago; it was later found that it also acts as an anticancer agent adjuvant (72). The combination of this drug with temozolomide (an imidazole derivate and a second-generation alkylating agent) promotes FZd-1 and GSK3-β phosphorylation (inactivated form) to stimulate β-catenin degradation and increase in the LC3 autophagy marker. Thus, thioridazine is proposed as a drug to increase P62-mediated autophagy by WNT/β-catenin signaling in glioblastoma cells, nonetheless, the combination with temozolomide exerts a better synergistic effect (73).

Lapatinib is an EGFR/HER2 tyrosine kinase inhibitor, administered orally for breast cancer and other solid tumors; it was approved by the FDA in 2007 (74). Lapatinib induced autophagy in cutaneous squamous cell carcinoma via mTOR inhibition and, at the same time, decreased Wnt/β-catenin expression, leading to EMT blockade. Although not purposely seeking a mechanistic interaction between Wnt signaling and autophagy, the evidence showed that the anticancer effect of lapatinib stems in the suppression of Wnt β-catenin signaling coupled with induction of autophagy (75). Niclosamide, Thioridazine, and Lapatinib mediate the disruption of the balance between WNT/β-catenin and autophagy; specifically, these drugs blocked Wnt/β-catenin and activated autophagy (73, 76).

Tao et al. reported that the activation of Wnt/β-catenin pathway with WNT3a inhibited autophagy increasing gemcitabine-induced apoptosis. Gemcitabine is a nucleoside antimetabolite that inhibits DNA synthesis approved for medical uses in 1995. Thus, this data suggested that Wnt/β-catenin activation sensitizes resistant osteosarcoma cells to gemcitabine by downregulating Beclin 1, an activator of autophagy (29). All these drugs have been tested in humans, which would facilitate their use in the clinic (Table 1).

## Concluding Remarks and Future Perspectives

In the literature reviewed, we found a list of targeted therapies employed as anticancer strategies to block WNT/β-catenin and autophagy pathways (Figure 1). So far, these targeted therapies only have been analyzed in *vitro*, in preclinical studies, and in early clinical trials, none of which has been approved yet (46, 66–70). This raises questions as to why there are no clinical trials in more advanced phases. Since the toxicity of these drugs remains an issue with an unclear answer (77), resorting to the use of other previously authorized drugs gains importance. In this regard, we found data about the inhibition of these pathways with drugs originally approved for other diseases, which brings WNT/β-catenin and autophagy targeting to the realm of drug repositioning (Figure 1).

Thus, due to the lack of advanced clinical trials using targeted therapies that inhibit WNT/β-catenin or autophagy, we think that the use of repositioned drugs can be a suitable therapeutic option to mediate the interplay between the two pathway in favor to anticancer effect (42, 43). We found that in most studies where WNT/β-catenin was pharmacologically inhibited, autophagy was promoted, demonstrating that both pathways are negatively correlated. Moreover, it appears that promoting autophagy is beneficial as anticancer mechanism. This strongly suggests that, in the current scenario, the soundest approach is to inhibit WNT/β-catenin signaling and to take advantage of its interplay with autophagy to purposefully increase it, bolstering the anticancer effect.

Interestingly, in the reviewed studies, the tested repositioned drugs, such as niclosamide and thioridazine, were originally designed for other diseases or were anticancer drugs such as lapatinib (71, 73, 75) and repurposed for cancer treatment. Currently, there are three clinical trials in phase I (ClinicalTrials.gov Identifiers NCT03123978, NCT02687009, and NCT02532114), and two in phase II (ClinicalTrials.gov Identifiers NCT02807805 and NCT025195829 recruiting patients to be treated with niclosamide; and only 1 in phase 1 using thioridazine (ClinicalTrials.gov Identifier NCT020962899). Further analyses are needed, so it will be beneficial to propose clinical trials involving cancer patients to be treated with niclosamide, thioridazine, and other repurposed drugs.

Considering all these data, we are confident that drug repurposing is the right path to follow given the absence of drugs that block pathways significant for tumor development, such as WNT/β-catenin and autophagy. Additionally, it is critical to determine whether inhibition of the Wnt/β-catenin pathway is relevant in itself or as a means to promote autophagy and thus contribute to the inhibition of tumor growth. Since the role of autophagy in cancer is still controversial, further exploration is necessary if we are to find a drug synergy that exploits the balance between Wnt/β-catenin signaling and autophagy. Drugs that impact on the interplay of both pathways—even if they are approved by the Food and Drug Administration for other diseases—are strong candidates for repurposing toward cancer therapy, and the possibilities are plentiful given the vast array of already-approved drugs. Research for drug repositioning is not new but, without a doubt, we are living a very exciting time in translational research.

## Author Contributions

AC-P conceived the review. CP-P, AC-P, and EL-U wrote the manuscript. CL-C contributed substantially over discussion and revision of the manuscript. ST-C prepared the figure. ST-C and V-GC contributed significantly in the search for information. All authors contributed to the article and approved the submitted version.

## Conflict of Interest

The authors declare that the research was conducted in the absence of any commercial or financial relationships that could be construed as a potential conflict of interest.
